# Observations on Ship Fever

**Published:** 1847-09

**Authors:** 


*Observations on Ship Fever.* By THOMAS BARBOUR, M. D., Att. Phy. to the City Hospital, St. Louis, Mo.—During the last two months of my attendance on the Charity Hospital of this city, about 130 Irish emigrants have been admitted, of which number about 100 have been affected, in various degrees, with what is termed ship fever. In about half of the cases, the disease existed only in its forming stage, manifested by great prostration of strength, loss of appetite, pallor of countenance, and more or less derangement of the digestive organs. In all the rest, the disease was fully developed on admission, and a majority of them presented a combination of the *worst* symptoms of typhus gravior.

The subjects of this affection left their native land, greatly enfeebled by long continued privation, and during a long voyage to this land of plenty, were crowded together in ill ventilated, and doubtless filthy apartments, with a meagre allowance of food and water. It is not surprising that, under such circumstances, a malignant form of fever should have been generated.

There is a diversity of opinion among the learned doctors here, relative to the nature of this fever, and to the question, whether it be contagious or not; some have pronounced it to be a bad form of typhoid fever. Most, I believe, entertain fears that it is propagable by contagion. As I design to be very brief, I will not stop to discuss these points, but will proceed to state such facts as have come under my observation, from which I think the following deductions may be fairly made :—First, that it is as genuine typhus fever as ever prevailed in any country; and secondly, that with proper attention to cleanliness and ventilation, no fears need be felt on the score of contagion.

The following symptoms, in a greater or less degree, marked all of the fully developed cases :—

Great prostration of strength; coolness of the extremities; flushed and rather livid cheek; small, weak, and frequent pulse; dryness and harshness of skin, at first, followed by cold and clammy sweat; tongue dry, red on edges, and brown or black, and cracked on the upper surface; dark incrustation of the teeth; insatiate thirst; excessive irritability of stomach; frequent serous, mucous or bloody discharges from the bowels; abdominal tenderness. In the worst cases, tympanitis, and constant singultus; more or less stupor, in many delirium; eyes dull, heavy, and suffused; in some cases, signs and symptoms of bronchitis and pneumonia; peculiar exanthema over the whole body in almost all; and, in one instance, well defined purpura hemorrhagica.

Treatment.—In the first instance, all the patients were put into a tepid bath, after which, in all the milder cases, in which the brain was not seriously implicated, I prescribed the following R. :—Hydrarg. prot. chlorid., grs. x; opii, grs. iv; ft. pil. iii, to be taken at once. In ten or fifteen hours after the above, I invariably ordered the following R. :—Mass. hydrarg. sulph. quinæ *aa* grs. xxx; sulph. morph., gr. i; ft. pil. xvi; two to be given every three hours. When the stomach was very irritable, or if there was abdominal tenderness, a blister of large size was applied over the epigastrium, or over the whole abdomen. If the skin was dry and harsh, a dessert-spoonful of spirit mindereri was given every hour or two, and mucilaginous drinks, as gum Arabic water, or flaxseed tea, were freely allowed. In some instances, the pills of calomel and opium were administered twice or three times, at intervals of twenty-four hours. In all the combination of quinine, blue pill and morphia was continued until convalescence was decided, then the solution of quinine with morphia, or quinine with Dover's powder, was administered, to prevent relapse.

In the second grade of the disease, in which the mucous membrane of the stomach and bowels appeared to be chiefly involved, the brain sympathizing in a greater or less degree, we prescribed calomel and opium, as above, and repeated and followed it with the pills of quinine, blue mass and morphia, or the following R. :—Hydrarg. prot. chlor., sulph. quinæ. *aa* grs. xx ; pulv. Doveri, grs. xxx ; ft. pulv. vi ; one to be given every three hours ; applied a large blister over the abdomen, and allowed the free use of mucilaginous drinks. When the alvine discharges were bloody, we prescribed as follows :—R. Hydrarg. prot. chlorid., acet. plumb., *aa* grs. xx ; opii. grs. x ; ft. pulv. vi ; one to be given every three hours. As soon as the bloody discharges ceased, we ordered the pills of blue mass, quinine and morphia, or the following :—R. Hydrarg. prot. chlorid., sulph. quinæ, camphor, *aa* grs. xx ; pulv. Doveri, 1 drachm ; ft. pulv. vi ; one to be given every three or four hours. When convalescence was decided, we prescribed the following :—R. Sulph. quinæ, grs. xxx ; sulph. ferri, 1 drachm ; aq. oz. iv ; ft. sol. A dessert-spoonful ter die. When tympanitis or singultus existed, we prescribed the following R. :—Tinct. assafœtid., spirit terebinth, *aa* 1 oz ; tinct. opii acet., 2 drachms ; m., a dessert-spoonful to be given every three or four hours. In some of the cases, the bowels could not be controlled by any of the above means, the patient being greatly prostrated by frequent discharges ; under these circumstances, we gave the following mixture :—R. Hydrarg. c. creta, 2 drachms ; acet. plumb., grs. xxx ; gum Arabic, ½ oz ; tinct. kino, 1 oz., tinct. opii acet., 2 drachms ; aq. oz. iv ; m. ; a dessert-spoonful to be given every three or four hours. In one case, after all the means detailed proved unavailing, we succeeded with the following R. :—Argent. nit., opii, *aa* grs. x ; ft. pil. x ; one to be given every four hours.

When decided symptoms of bronchitis or pneumonia existed, in addition to the general treatment already detailed, we ordered the chest to be cupped, applied a blister, or croton oil, and administered the following R. :—Carb. ammon., ext. hyosciam. *aa* grs. xx ; antim. tart., grs. ii ; gum Arabic, ½ oz ; mist. camph., oz. iv ; m. ; a dessert-spoonful to be given every two or three hours.

In those cases in which the brain appeared to be seriously implicated, indicated by stupor or delirium, we ordered blood to be taken, by cups, from the nucha, and applied a blister, 3 by 6 inches, to the upper portion of the spine, and administered calomel and opium, as already prescribed, and followed it with the pills of blue mass, quinine and morphia, together with such other means as were indicated, on account of pulmonic or abdominal complications.

So great was the tendency to collapse, in most of the cases, from the time of admission, that we deemed it proper to allow the free use of toddy, burnt brandy, or milk dashed with brandy.

I am aware that the above treatment (I refer particularly to the large doses of opium and the use of quinine,) is totally opposed to the very best authorities in Europe and America, but it must be conceded that the results of my practice contradict all authority upon this subject, when I candidly assert that all of the milder cases rapidly recovered, and that of about fifty cases, all fully developed, and most of them of a malignant grade, only six have died, of whom three were so low at the time of admission, that but little hope was entertained of their recovery, under any treatment. Those now remaining in the Hospital are all decidedly convalescent.

I do not doubt but that my views of treatment will be condemned by many who may read this *hastily* written sketch, and especially by the older members of the profession, most of whom are averse to leaving the old beaten track that has been trod for centuries by our illustrious predecessors; but I indulge the hope that the above practical observations may excite a spirit of inquiry in the minds of unprejudiced young men, which may eventually convince them of the *invaluable truth*, that, except under circumstances of cerebral inflammation, in which depletion, purgatives, revulsives, &c., should be unquestionably premised, full doses of opium and quinine, *judiciously used*, are the most certain and efficient weapons of defence against essential fever, the deadliest enemy to man, under whatever garb it may be presented.

In conclusion, I would remark that, among the numerous patients in the Hospital, at the time of admission, of the cases of ship fever, and who have been not only in the same wards, but in contiguous beds with the fever patients, not one has contracted the disease.—*Missouri Medical and Surgical Journal.*

